# A novel, simple and rapid route to the synthesis of boron cabonitride nanosheets: combustive gaseous unfolding

**DOI:** 10.1038/s41598-017-03794-7

**Published:** 2017-06-14

**Authors:** Maisam Jalaly, Francisco José Gotor, Masih Semnan, María Jesús Sayagués

**Affiliations:** 1 Nanotechnology Department, School of New Technologies, Iran University of Science & Technology (IUST), Narmak, Tehran, 16846-13114 Iran; 2 Instituto de Ciencia de Materiales de Sevilla (CSIC-US), Americo Vespucio 49, Sevilla, 41092 Spain

## Abstract

The ternary compound boron carbonitride (BCN) was synthesized in the form of few-layer nanosheets through a mechanically induced self-sustaining reaction (MSR). Magnesium was used to reduce boron trioxide in the presence of melamine in a combustive manner. The process to form the nanostructured material was very rapid (less than 40 min). The prepared powder was investigated by various techniques such as X-ray diffraction (XRD), Fourier Transform infrared (FTIR), Micro-Raman spectroscopy, X-ray photoelectron spectroscopy (XPS), high-resolution transmission electron microscopy (HRTEM), and electron energy loss spectroscopy (EELS). The thermal stability and the optical behavior of the BCN nanosheets were also studied by thermal analysis and UV-vis spectroscopy, respectively. The formation mechanism of the nanosheet morphology was described in detail.

## Introduction

Hexagonal boron nitride (BN) is a well-known insulator and has a layered graphitic structure with strong bonds within atomic layers and weak bonds between layers^1^. The outstanding properties of BN, including its chemical inertness, thermal stability, high melting point, low density, high thermal conductivity and good optical transparency, make it an attractive candidate for various applications in the thermal, optical, and electronic industries^2^. Incorporation of carbon atoms into BN lattice creates a three-component material that can intensify mechanical properties of BN and convert it from an insulator to a semiconductor. Ternary compounds containing boron, nitrogen and carbon elements have attracted great attention due to their potentially remarkable properties. Boron carbonitride may also appear in either cubic BCN (c-BCN) or hexagonal BCN (h-BCN) forms. c-BCN can exhibit the advantages of diamond and c-BN behaviors concurrently, while h-BCN is expected to behave as a semiconductor due to its band gap energies^3, 4^.

Nanoscale BCN has been synthesized in various nanostructured forms such as nanowires^5^, nanofibers^6^, nanosheets^7–10^, nanotubes^11–17^, spherical nanocages^2^, hollow spheres^18^, mesoporous^19^, and amorphous^20, 21^ structures. To synthesize these various BCN nanostructures, several methods have been employed depending on the desired morphology of the product. An elemental substitution reaction under nitrogen-containing atmospheres at high temperatures using a 3D mesoporous carbon template has been used to create different mesoporous products^2, 19^. High temperature nitridation of boron carbide (B_4_C) under different pressures of N_2_ gas has also been tested^3^. A BCN material containing a small amount of oxygen and hydrogen impurities was produced using a liquid-based solvothermal technique^4^. There are numerous reports on the usage of chemical vapor deposition (CVD) as a gas-state technique^5, 6, 10–17^. By using vapor-phase pyrolysis of polymeric precursors, crystalline hollow spheres^18^ and amorphous^20, 21^ BCN were synthesized. Long-term mechanical milling of a mixture of commercial BN and graphite was also used to form a ternary BCN materials^22–24^. As can be deduced from the literature, CVD-grown one-dimensional (1D) structures of BCN have attracted the most research attention, and there are few reports on 2D freestanding structures.

Similar to graphene, hexagonal BN and hexagonal BCN can have 2D natures and can be fabricated as single- or few-layered nanosheets. Thus, these materials are sometimes called graphene analogues. Recently, BCN nanosheets were synthesized using graphene oxide as the sheet-like starting material along with boron oxide and solid/gaseous nitrogen-containing species at high temperatures under controlled atmospheres^7, 8^. Ahmed and co-workers^9^ used a mixture of boric acid, lactose and urea to synthesize BCN nanosheets through a solid-state reaction at high temperature (1200 °C for 2 hours). A gas-state CVD process from a mixture of BF_3_–CH_4_–N_2_–H_2_ was also accomplished by Qin *et al*. to produce 2D BCN sheets^10^.

Recently, the metallothermic reduction of oxide ceramics has been employed to produce valuable non-oxide engineering ceramics^25–27^. This type of reaction can progress in the self-propagating high-temperature synthesis (SHS) regime, which is also called the combustion reaction. Using mechanical milling, chemical reactions can also be induced through the mechanical energy supplied by ball impacts. This method for triggering reactions is called mechanosynthesis. Mechanochemical processes can be referred to as mechanically induced self-sustaining reactions (MSRs) when an SHS reaction is induced by the high-energy ball milling of the reactants.

Although melamine has been frequently used as a strong nitrifying agent in synthesizing nitride^28–30^ or carbonitride^31, 32^ compounds, heat treatment at relatively high temperatures for a given duration was often used to complete the reaction. In most of these studies, melamine reacts directly with metal oxides or elemental metals to form the nitride products. However, there are no reports on the participation of melamine in a metllothermic combustion reaction to synthesize a carbonitride compound with a nanosheet structure. Therefore, in the present work, we focused on the reduction of boron trioxide by magnesium in the presence of melamine additive (as a solid nitrogen-containing organic compound) through a solid-state combustion reaction that is a novel process for synthesizing boron carbonitride. The idea behind this procedure was to use the combustive nature of the reduction of boron oxide by magnesium to stimulate melamine breakdown into carbon- and nitrogen-containing species to synthesize boron carbonitride.

## Experimental

The raw Mg (99%, Daejung, South Korea), B_2_O_3_ (98%, Merck, Germany), and melamine (99%, SamChun, South Korea) powders were used as-purchased without any extra purification to produce boron carbonitride powder. Since boron oxide can readily adsorb the atmospheric moisture, it was always kept inside the oven at 100 °C to prevent adsorbing undesired moisture. The starting materials were mechanically milled in a planetary high-energy ball mill (Model: NARYA, Amin Asia Fanavar Pars Co., Iran) under an argon atmosphere. The ball milling process was performed with a ball-to-powder mass ratio (BPR) of 40:1 and a rotational speed of 600 rpm. About five grams of powder together with eleven chromium hardened steel balls (10, 15 and 20 mm in diameter) were placed in a hardened steel vial (250 ml) for each milling experiment. To explore the ignition onset time, the temperature of the milling vial was measured using a thermocouple attached to its external wall. The appearance of a temperature rise implies the occurrence of the MSR reaction, and hence, the ignition time can be determined. Any magnesium oxide by-product was removed by leaching the as-milled powder in a 1 M HCl solution at 80 °C for 1 h.

X-ray powder diffraction (XRD) patterns of the samples were acquired with a PANalitycal X’Pert diffractometer (Almelo, the Netherlands) (45 kV, 40 mA) with Cu Kα radiation (λ = 0.15406 nm). Data were collected in the range of 10–90° (2θ) in step-scan mode with a step of 0.03 and a counting time of 1 s/step. Transmission Electron Microscopy (TEM) images were obtained in a 200 kV Philips CM200 microscope (FEI Europe, Eindhoven, the Netherlands) equipped with a superTWIN objective lens and an LaB_6_ filament (point resolution 0.25 nm). High-resolution transmission electron microscopy (HRTEM) was performed with a 300 kV TECNAI G2 F30 microscope (FEI Europe) equipped with a field emission system (point resolution 0.2 nm), an SDD X-Max EDS detector (Oxford Instruments, Abingdon, UK) and a GIF Quantum SE filter (Gatan Inc., Pleasanton, USA) for electron energy loss spectroscopy (EELS). The analysis of the HRTEM images and the EELS spectra was performed with the Digital Micrograph software (Gatan Inc.). The samples were prepared by dispersion of the powder in acetone, and droplets of the suspension were deposited onto a holey coated carbon copper grid.

Raman spectroscopy was performed using a dispersive Horiba Jobin Yvon HR800 confocal Raman Microscope (HORIBA, Kyoto, Japan) equipped with a charge-coupled device (CCD) detector at a laser excitation wavelength of 532 nm (green laser). The spectral resolution was 4 cm^−1^. The laser beam was focused on the sample with a confocal objective of 100×. FTIR spectra were recorded in a Jasco FTIR 6200 IRT-5000 spectrometer (Jasco Inc., Easton, USA). Spectra were collected at 4 cm^−1^ resolution in transmission mode (samples diluted 1/100 in KBr). X-ray photoelectron spectroscopy (XPS) studies were carried out on a Leybold-Heraeus LHS-10 spectrometer, working with constant pass energy of 50 eV. The spectrometer main chamber, working at a pressure < 2 × 10^−9^ Torr, was equipped with an EA-200 MCD hemispherical electron analyzer with a dual X-ray source working with Mg Kα (hν = 1253.6 eV) radiation at 120 W and 30 mA. The C 1 s signal (284.6 eV) was used as the internal energy reference in all the experiments. Samples were outgassed in the pre-chamber of the instrument at 150 °C up to a pressure < 2 × 10^−8^ Torr to remove chemisorbed water. All photoelectron spectra were analyzed using Spectral Data Processor (SDP) software. The thermal behavior of the powders was studied by differential thermal analysis (DTA) and thermal gravimetry (TG) in a TA Instrument Q600 (New Castle, DE) using a constant heating rate of 10 °C/min from room temperature to 850 °C. The solid-state diffuse reflectance spectra (DRS) of the samples in the wavelength range of 200–750 nm were obtained by a Shimadzu UV–vis spectrophotometer (Kyoto, Japan) with BaSO_4_ as the standard reference. The band gap energy was calculated according to the Tauc method.

## Results and Discussion

### Synthesis aspects

Different amounts of melamine, with chemical formula of C_3_N_6_H_6_, (10, 16, and 23 wt.%) were added to the stoichiometric mixture of boron oxide and magnesium according to the following reaction:

1 $$3{\rm{Mg}}+{{\rm{B}}}_{2}{{\rm{O}}}_{3}\to 2{\rm{B}}+3{\rm{MgO}}$$

$${{{\rm{\Delta }}{\rm{G}}}^{^\circ }}_{298}=-515\,{\rm{kJ}},{{{\rm{\Delta }}{\rm{H}}}^{^\circ }}_{298}=-532\,{\rm{kJ}},{{\rm{T}}}_{{\rm{ad}}}\approx 2630\,{\rm{K}}$$

The samples containing 10, 16 and 23 wt.% melamine are hereafter designated as M10, M16 and M23, respectively. As seen, the reaction 1 is very exothermic and possesses a large adiabatic temperature (T_ad_). We have shown in a previous work^33^ that, under severe milling conditions, Mg and B_2_O_3_ in a binary system react with each other in a combustion regime after a short time of milling (approximately 27 min) and produce amorphous elemental boron. In the current work, the ternary mixture of initial materials was subjected to similar milling conditions. The combustion reaction occurred again after a short milling time for each sample (30, 35 and 40 min of milling for M10, M16 and M23 samples, respectively). In fact, the initial materials are mechanically activated during these periods and absorb the energy required to pass the energy barrier of the reaction and to cause the combustion. The ignition time was observed to increase with melamine content.

**Figure 1:**
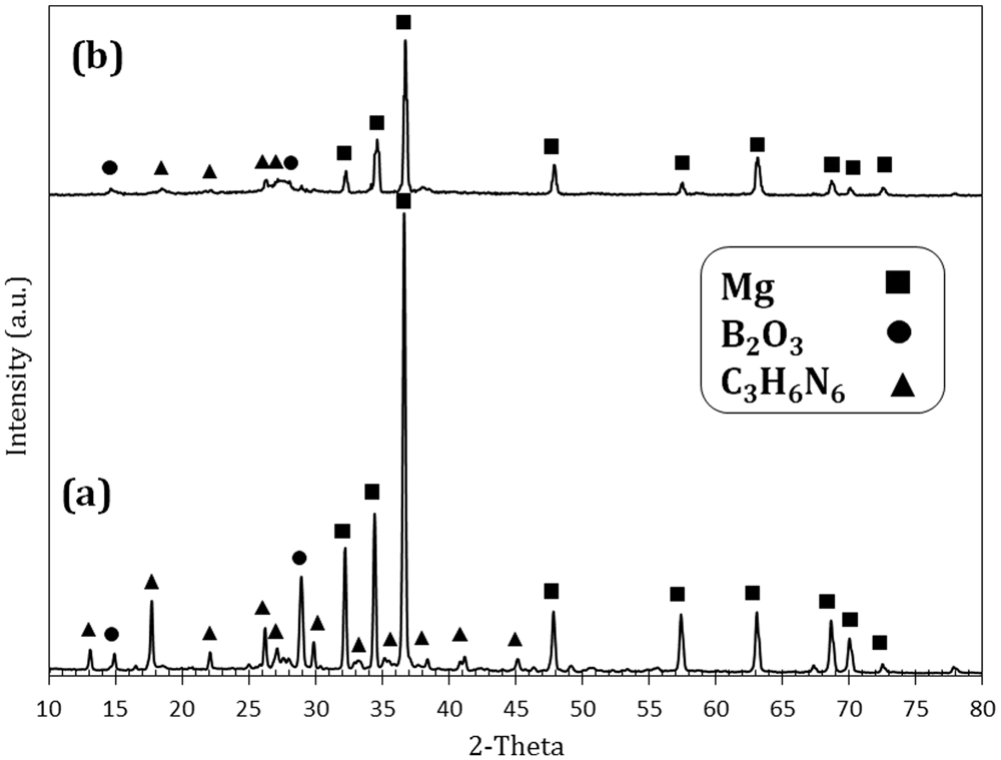
X-ray diffraction patterns of the (**a**) initial mixture and (**b**) 15 min-milled powders in M10 sample.

**Figure 2:**
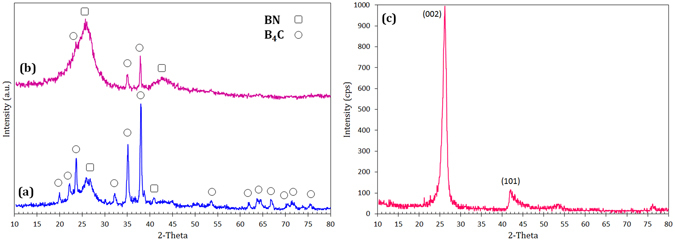
X-ray diffraction patterns of the (**a**) M10, (**b**) M16, and (**c**) M23 samples after combustion and the leaching treatment.

Figure 1 shows the XRD patterns of initial mixture and 15 min-milled sample for M10 system. Mg (ICDD PDF #35-0821), B_2_O_3_ (ICDD PDF #06-0297), and melamine (ICDD PDF #39-1950) are observed in the initial mixture. As a typical sample before combustion, M10 milled for 15 min was shown (Fig. 1(b)) to be composed of only un-reacted initial materials with lower intensities, suggesting that the materials were being activated. After combustion, the milled powders were collected and an acidic leaching treatment was performed (see Methods section) to remove the MgO by-product. Figure 2 shows the products of the samples with different amounts of melamine after combustion and leaching process. It can be observed that the M10 and M16 samples are composed of both hexagonal boron nitride (BN, ICDD PDF #35-0798) and boron carbide (B_4_C, ICDD PDF #85-1068) and that the sample M23 is free of B_4_C. In fact, Fig. 2 indicates that as melamine content in the starting mixture increases, the amounts of boron carbide and boron nitride decrease and increase, respectively, such that finally, sample M23 becomes a single phase and is free of the carbide phase. The high intensity of the XRD peaks of the sample M23 can be considered a clear indication for high crystallinity of the product. In Fig. 2(b), two representative XRD peaks centered at approximately 2*θ* = 26.1 and 41.9° were observed, which is very similar to the (002) {2*θ* = 26.6°} and (101) {2*θ* = 41.5°} planes of h-BN phase. This slight shift relative to the standard h-BN phase might be attributed to the inclusion of carbon atoms within the BN lattice to form a carbonitride phase. A similar shift in the XRD peaks of BCN relative to those of BN and graphite has also been observed by other groups^3, 4, 9, 20^. From the XRD pattern, the interplanar spacings for two major planes of the lattice (*d*_002_ and *d*_100_) and the lattice parameters of *a* and *c* were calculated to be 3.403, 2.153, 2.486, and 6.806 Å, respectively. The calculated *d*-spacing of *d*_002_ is slightly larger than that of standard ASTM h-BN (3.33Å^34^). The calculated value of 2.153 Å for *d*_100_ is also between the reported values corresponding to h-BN (*d*_100_ = 2.17 Å) and graphite (*d*_100_ = 2.13 Å)^9^. Using the Williamson-Hall equation^35^, the crystallite size of sample M23 was estimated to be approximately 6.9 nm. In this method, a standard silicon sample was employed to eliminate the effect of instrumental broadening.

**Figure 3:**
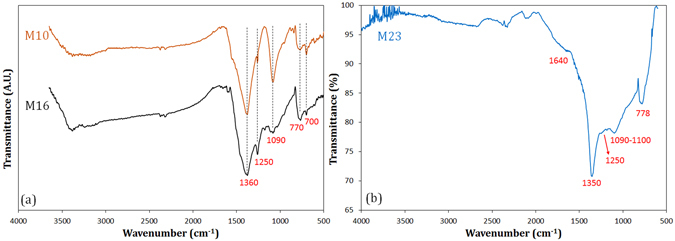
FTIR spectrum of sample M23 after combustion and the leaching treatment.

To provide a deeper insight into the chemical structures of the materials and to confirm that the product of the synthesis in sample M23 is BCN and not BN, several complementary analyses were performed. FTIR analysis was performed for M10, M16, and M23 samples and the results are shown in Fig. 3. The FTIR spectra of the composite samples of M10 and M16 (Fig. 3(a)) show B–N bond at 770 and 1360 cm^−1^ and B–C bond at 700, 1090, and 1250 cm^−1^ ^36–39^. As seen, the peaks related to B–C bond have become smaller with increasing melamine content, indicating a decrease in B_4_C amount for higher amount of melamine. This is consistent with the XRD results. The decrease of B–C bond is clearly observed for sample M23 which is free of carbide phase according to the XRD analysis. The FTIR spectrum of M23 sample was separately depicted in Fig. 3(b) for better interpretation. It shows five characteristic bands located at approximately 778, 1090, 1250, 1350 and 1640 cm^−1^. The large absorption band at ~1350 cm^−1^ and the small band at ~778 cm^−1^ are attributed to the in-plane stretching vibration and out-of-plane bending vib;ration of B–N and B–N–B bonds, respectively^37, 38^. The band at approximately 1090 cm^−1^ corresponds to both C–N and B–C bonds, and the bands located at 1250 and 1640 cm^−1^ can be assigned to the B–C and C–N, and C=N bonds, respectively^38–40^. The band nominally at 1090 cm^−1^ has a somewhat broad tip extending from 1090 to 1100 cm^−1^. The feature at ~1100 cm^−1^ can be related to the asymmetric stretching vibration of B–O bond^41^, which indicates a very small amount of surface oxidation. No C–H and N–H bonds were observed in the FTIR pattern, suggesting no starting melamine remained after combustion. The appearance of such bonds in the FTIR spectrum reveals that the material synthesized by the combustion is a B–C–N material. It can also be deduced from the low intensities of bonds including carbon component that there is a low carbon content in the BCN structure.

**Figure 4:**
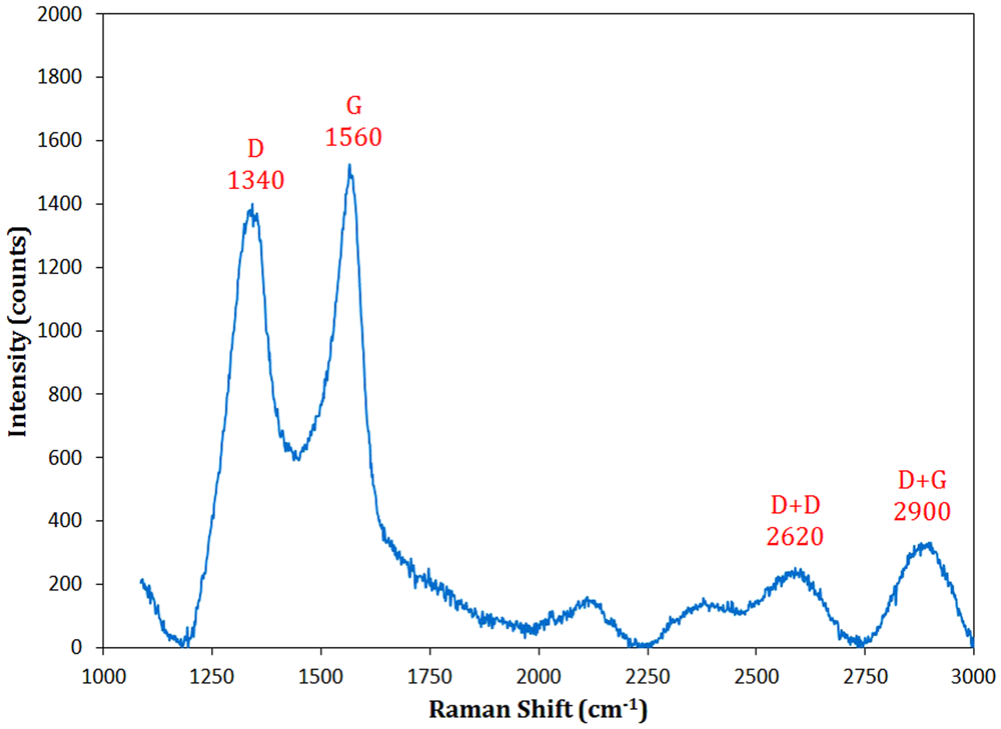
Raman spectrum of the combusted BCN after combustion and the leaching treatment.

Raman spectroscopy is a popular, non-destructive technique for detecting structural information of layered materials such as graphene and BN. The representative Raman spectrum of sample M23 is shown in Fig. 4. Boron nitride material displays a typical single Raman peak, whose position and shape are dependent on its structure and layers (~1366 cm^−1^ for bulk BN)^42–44^, whereas BCN nanomaterials display separated D and G bands^11, 45, 46^. The Raman spectrum of M23 shows two intense, broad peaks centered at approximately 1340 and 1560 cm^−1^ and two small, broad peaks centered at approximately 2620 and 2900 cm^−1^. There is a close resemblance between the first two bands and the first-order D and G bands of graphite, respectively^47^. These two characteristic bands are usually assigned to the disordered (D) and graphitic (G) nature and represent the graphitic structure of the material. The G peak originates from the first-order scattering from the doubly degenerate E_2g_ phonon mode as well as bond stretching of all sp^2^-hybridized pairs in both rings and chains (such as C–C, B–C, C–N, and B–N bonds). Moreover, the D peak is due to a breathing mode of A_1g_ symmetry in aromatic rings^47, 48^. The full width at half maximums (FWHMs) for D and G peaks are ~120 and 100 cm^−1^, respectively, which are significantly broad peaks. This broadening may be ascribed to the substitution of boron and nitrogen atoms with carbon into BCN material and formation of different bonds (such as B–C, C–N, and B–N) with different bond lengths and masses, resulting in a distortion in the graphitic layers^11^. The other peaks, located at ~2620 and 2900 cm^−1^, are called 2D (D + D) and D + G bands and correspond to the second-order of Raman spectra in overtone and combination modes, respectively. These combination peaks have also been observed in Raman characterization of BCN nanotubes^11, 17^. The intensity ratio of the major peaks, *I*_*D*_/*I*_*G*_, is found to be 0.91. The higher intensity of G band implies a higher degree of ordered graphitic nature of the BCN. It has been experimentally demonstrated that the ratio of *I*_*D*_/*I*_*G*_ has an inverse relation with the lateral crystallite size (*L*_*a*_). The following empirical equation was presented for this correlation^47, 48^:

2 $${L}_{a}\approx 4.4{(\frac{{I}_{D}}{{I}_{G}})}^{-1},{\rm{nm}}$$

The lateral crystallite size of nanostructured BCN was calculated to be approximately 4.8 nm, which is in close agreement with the crystallite size calculated by XRD data (6.9 nm). Based on the Raman analysis, it can be concluded that sample M23 is a layered graphite-like BCN compound.

**Figure 5:**
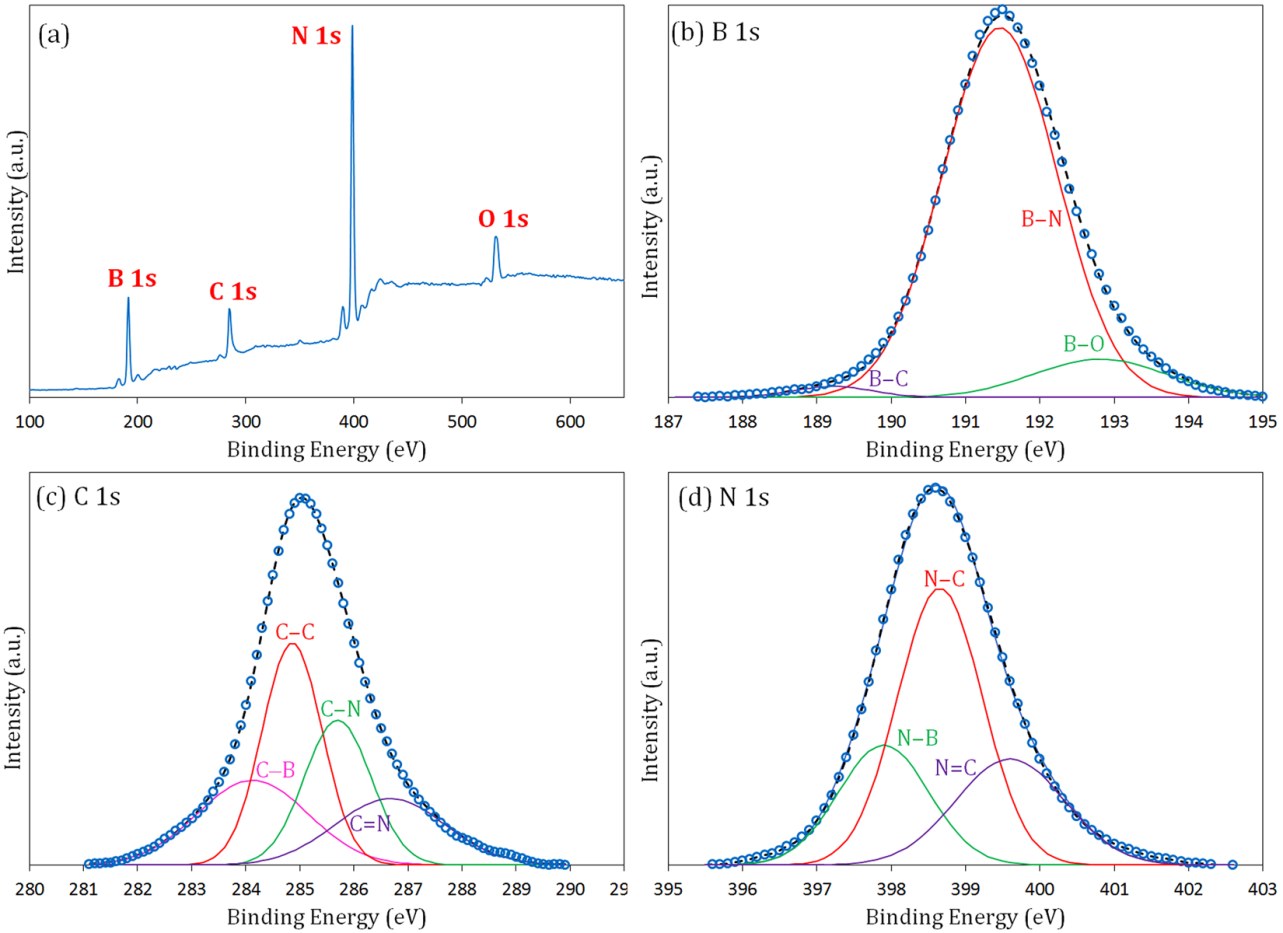
XPS data of sample M23 after combustion and the leaching treatment (open circles). Full survey scan (**a**) and high resolution spectra together with deconvoluted peaks for B 1 s (**b**), C 1 s (**c**) and N 1 s (**d**). The dashed lines are the resulting curves obtained by Gaussian fitting of the deconvoluted curves.

To further investigate the chemical nature of the prepared powder, XPS analysis was performed on sample M23. Figure 5 shows the survey scan together with high resolution spectra of B 1 s, C 1 s, and N 1 s. The spectrum of the full survey scan displays the presence of boron, carbon, nitrogen and oxygen atoms in the sample. Therefore, the analysis substantiates the carbonitride, rather than nitride, nature of the material. The appearance of a small peak related to the oxygen seems to be due to the surface contamination (see EELS analysis) and is almost always observed in similar cases^4, 7, 17, 18^. The percentages of B, C, and N in the B_*x*_C_*y*_N_*z*_ nanomaterial were calculated from survey scan spectrum to be approximately 39%, 12%, and 42%, respectively, implying the chemical formula of BC_0.3_N_1.08_.

Deconvolution of B 1 s spectrum in Fig. 5(b) gives three peaks centered at 189.2, 191.3, and 192.8 eV. The major peak at 191.3 eV has been reported to correspond to the B–N bond^49, 50^, while the two other peaks at lower and higher energies are attributed to the B–C and B–O bonds, respectively^4, 7^. The FWHM of the B 1 s spectrum for our prepared BCN was measured to be ~1.8 eV, which is remarkably larger than that for pure h-BN (0.92^47^). This is an indication for the existence of additional atoms other than nitrogen surrounded boron atoms (like C and O). Based on Fig. 5(c), the C 1 s signal can be deconvoluted into four bands located at 284.0, 284.7, 285.7, and 286.7 eV, respectively. The largest peak at 284.7 and the peak at 284.0 eV are assigned to graphene-like C–C bonds^7, 8^ and C–B bonds^8^, respectively. The linkage between carbon and nitrogen atoms appeared in both sp^2^-type C=N and sp^3^-type C–N bond forms at energy values of 285.7 and 286.7 eV, respectively^8, 40^. The FWHM for C 1 s is ~2 eV, which is broader than that for graphite (0.35 eV^51^), indicating that carbon atoms are surrounded with various bonds. The result of deconvolution of N 1 s spectrum is shown in Fig. 5(d). The N 1 s data are composed of three components centered at 397.9, 398.7, and 399.6 eV, which are assigned to the N–B, N–C, and N=C, respectively^7–9, 18, 40^. The FWHM of the N 1 s spectrum was detected to be ~1.8 eV (wider than that for h-BN, 0.88^52^). The outcomes of XPS analysis are perfectly consistent with the bonds found by FTIR analysis. The overall XPS results suggest that all boron, carbon, and nitrogen atoms are atomically intermixed and are not separate in the forms of graphite and h-BN.

### Morphology and mechanisms

The mechanism underlying this process has three major stages: (1) combustive reduction of boron oxide, (2) decomposition of melamine, and (3) formation of boron carbonitride. The overall reaction (ternary system) was initially induced by the sub-reaction of Mg–B_2_O_3_, as shown elsewhere^25, 26, 33^. According to the adiabatic temperature (T_ad_) and enthalpy value of reaction 1, the required conditions to satisfy the empirical Merzhanov’s criterion (T_ad_ > 1800 K^53^) to proceed in a self-sustaining manner were fulfilled (this phenomenon was also observed in practice as reported). Thus, Mg reduces boron oxide in a combustive reaction to obtain elemental boron together with the release of a great amount of heat; this heat increases the system temperature inside the milling vial to such a level as to trigger the reaction of melamine decomposition. In fact, magnesium plays the major role of a strong reducing agent for our boron-containing source (B_2_O_3_) to provide nascent boron in a combustive manner in a short duration. After the onset of self-sustaining reaction induced by milling, a high temperature is provided by highly exothermic magnesiothermic reduction that gives rise to melamine decomposition and production of carbonitride phase. Melamine, C_3_N_3_(NH_2_)_3_, has a central *s*-triazine ring skeleton with three amine groups. It has been shown^54, 55^ that these amine groups (−NH_2_) are the weakest bonds in melamine molecule and gradually separate from the main skeleton during decomposition. In fact, melamine has been reported to undergo multiple condensation steps above ~550 °C, during which several intermediate compounds are formed as a consequence of successive deammoniation steps^55–58^. With increasing temperature, these intermediates, including melam {(C_3_N_3_)_2_(NH_2_)_4_(NH)}, melem {C_6_N_7_(NH_2_)_3_}, melon {(C_6_N_7_)_3_(NH_2_)_3_(NH)_3_}, and graphitic carbon nitride {g-C_3_N_4_}, are generated as follows:

3 $$2{{\rm{C}}}_{3}{{\rm{N}}}_{6}{{\rm{H}}}_{6}\to {{\rm{C}}}_{6}{{\rm{N}}}_{11}{{\rm{H}}}_{9}+{{\rm{NH}}}_{3}$$

4 $${{\rm{C}}}_{6}{{\rm{N}}}_{11}{{\rm{H}}}_{9}\to {{\rm{C}}}_{6}{{\rm{N}}}_{10}{{\rm{H}}}_{6}+{{\rm{NH}}}_{3}$$

5 $$3{{\rm{C}}}_{6}{{\rm{N}}}_{10}{{\rm{H}}}_{6}\to {{\rm{C}}}_{18}{{\rm{N}}}_{27}{{\rm{H}}}_{9}+3{{\rm{NH}}}_{3}$$

6 $${{\rm{C}}}_{18}{{\rm{N}}}_{27}{{\rm{H}}}_{9}\to 6{{\rm{C}}}_{3}{{\rm{N}}}_{4}+3{{\rm{NH}}}_{3}$$

At higher temperatures, g-C_3_N_4_ will decompose into reactive radical moieties such as C_2_N_2_^+^, C_3_N_2_^+^, and C_3_N_3_^+^^59^. It is worth noting that melamine has been demonstrated to be an effective nitrifying agent at lower temperatures and a suitable carbiding agent at higher temperatures^56, 58^. At lower temperatures, the carbon nitride (and/or its decomposed radicals) can react with reduced boron to form boron nitride phase as follows:

7 $${{\rm{C}}}_{3}{{\rm{N}}}_{4}\{{\rm{or}}\,{{\rm{C}}}_{x}{{{\rm{N}}}_{y}}^{+}\}+4{\rm{B}}\to 4{\rm{BN}}+3{\rm{C}}$$

At higher temperatures, this nitride phase can transform to the carbide phase^58^:

8 $${{\rm{C}}}_{3}{{\rm{N}}}_{4}\{{\rm{or}}\,{{\rm{C}}}_{x}{{{\rm{N}}}_{y}}^{+}\}+12{\rm{BN}}\to 3{{\rm{B}}}_{4}{\rm{C}}+8{{\rm{N}}}_{2}$$

A portion of ammonia gas generated in the reactions 3–6 can react with the initial boron oxide in the combustion moment to further progress the formation of BN as follows:

9 $${{\rm{B}}}_{2}{{\rm{O}}}_{3}+{{\rm{NH}}}_{3}({\rm{g}})\to 4{\rm{BN}}+{{\rm{H}}}_{2}{\rm{O}}\,({\rm{g}})$$

10 $${{\rm{H}}}_{2}{\rm{O}}({\rm{g}})+{\rm{C}}={\rm{CO}}({\rm{g}})+{{\rm{H}}}_{2}({\rm{g}})$$

In practice, boron carbide was the main product, along with a small amount of BN, when 10 wt.% melamine was used. The amount of B_4_C decreased as the starting amount of melamine was increased to 16 wt.%. Finally, boron carbide phase was eliminated by using a larger amount of melamine (23 wt.%). It can be deduced that melamine powder acts as a diluent for the combustive reaction of Mg–B_2_O_3_, resulting in absorption of a fraction of combustion energy by melamine being heated, and overall, the temperature will decrease. As mentioned above, the ignition time was slightly increased by increasing the melamine amount (27, 30, 35, and 40 min for 0, 10, 16, and 23 wt.% melamine, respectively). Additionally, the combustion reaction did not occur at all (even after some hours) when the amount of melamine increased to ~30 wt.% (not shown here). This evidence confirms the diluent role of melamine and ignition delay with increasing melamine quantity. Therefore, the melamine is an energy sorbent and lowers the temperature of combustion flame. The more melamine that is used, the lower the temperature that is obtained. This is the reason why there is no boron carbide compound among the combustion product in M23 sample, as the temperature has lowered to a level favoring only the formation of nitride phase (having only a little amount of carbon). According to the reaction 7, carbon exists with the boron nitride. Most of this carbon would leave the system due to its conversion into CO (reaction 10). A small amount of carbon can diffuse within the BN structure at the high temperature provided by the combustion, and therefore, the boron carbonitride phase is formed. The simultaneous presence of both the nitride and carbide phases in the products of samples M10 and M16 (with different fractions) can be explained by the very short ignition moment and an insufficient opportunity for the carbide-nitride conversion (i.e. incomplete progress of reaction 8).

**Figure 6:**
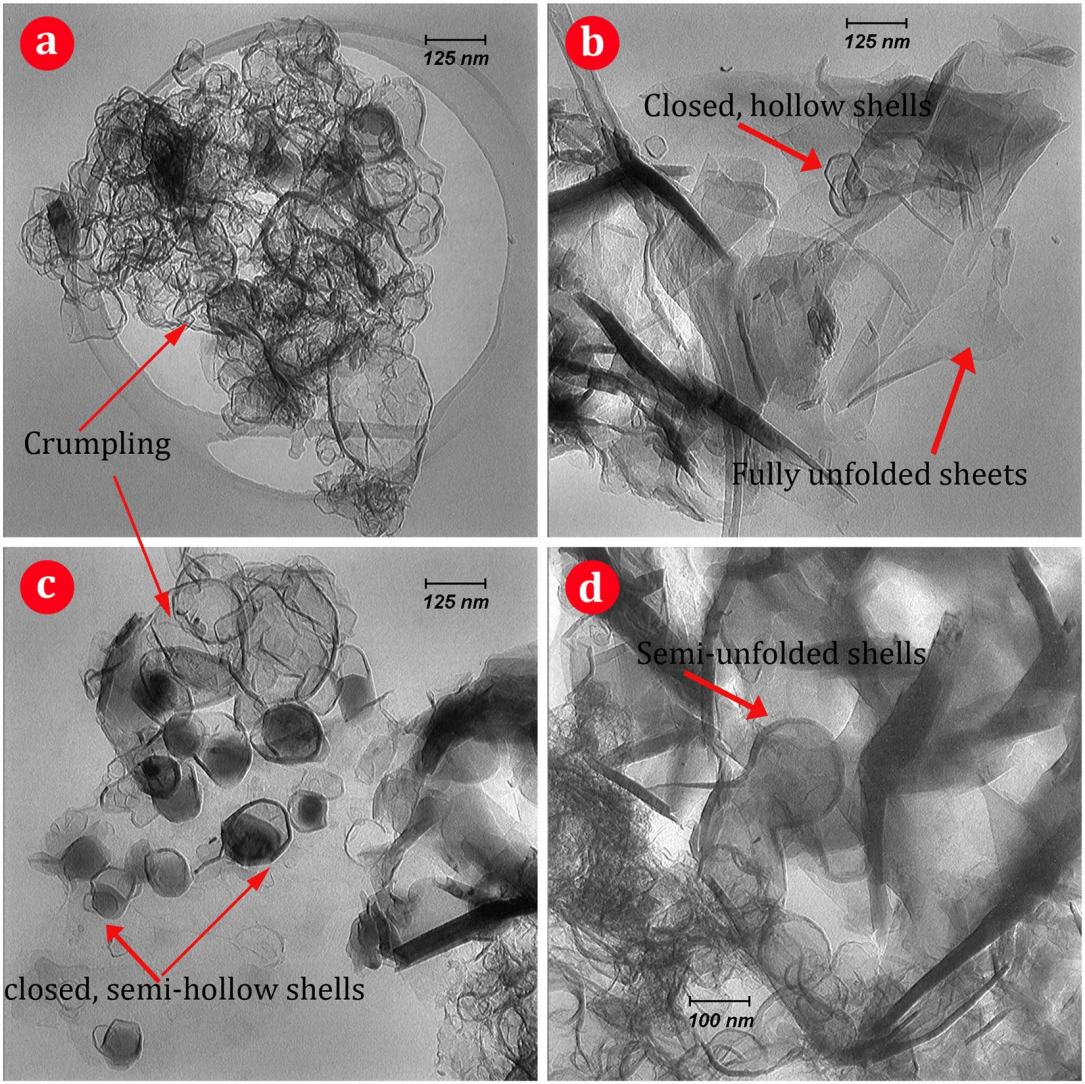
TEM images of the synthesized BCN nanosheets.

**Figure 7:**
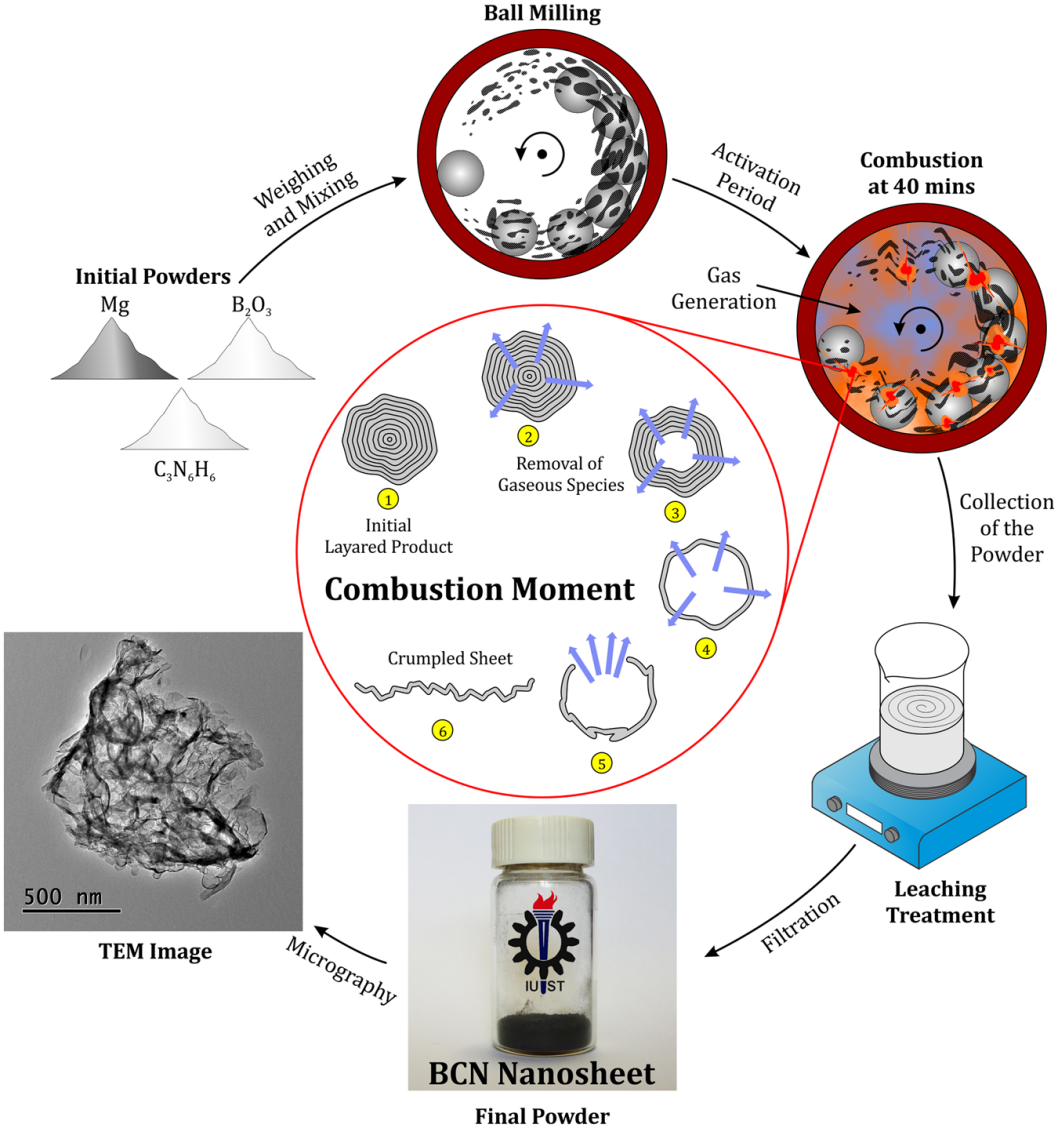
Schematic representation of the synthesis of BCN nanosheets.

**Figure 8:**
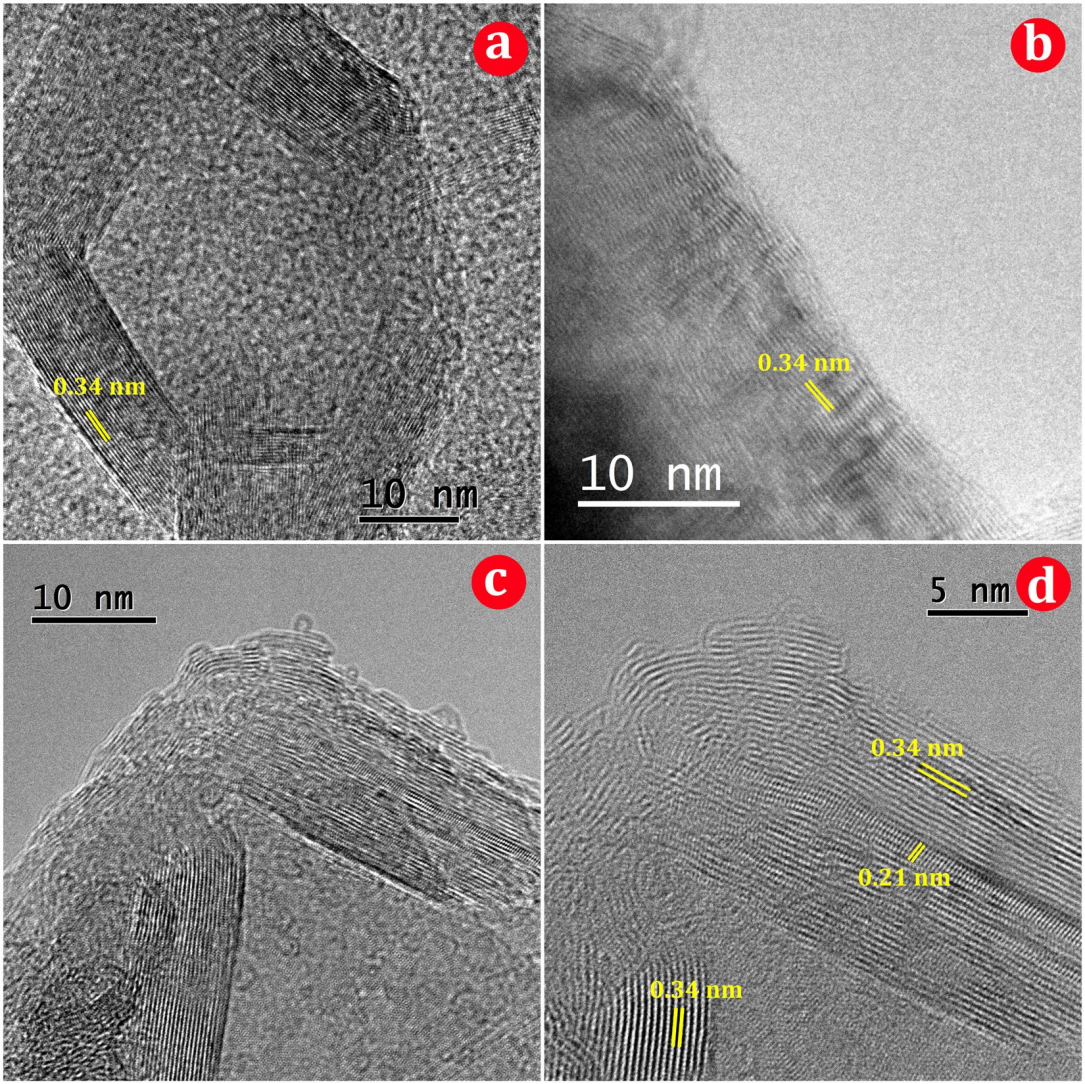
HRTEM images of the BCN nanosheets: (**a**) one hexagonal closed hollow shell, (**b**), (**c**), and (**d**) different positions in the edges of the nanosheets.

The morphology of nanostructured BCN was analyzed using TEM and HRTEM. The typical TEM micrographs depicted in Fig. 6 corroborate the synthesis of the BCN into thin nanosheets that resemble exfoliated 2D materials. As shown in the low magnification TEM image (Fig. 6(a)), nanosheets are stacked together, and a great deal of bending, curling and crumpling are observed in the structure, similar to graphene and BN nanosheets^60–62^. There are both highly crumpled and moderately crumpled points among the nanosheets. Additionally, most of the nanosheets exhibit folds near their edges (Fig. 6). The darker strips surrounding the sheets are folded edges and crinkles of the nanosheets. Formation of such a sheet-like morphology can be attributed to the nature and mechanism of the reactions involved in the process. As explained above, a combustive reaction occurs in a moment, and concurrently, melamine disassociates in several steps and generates a great deal of gaseous species (such as H_2_, CO and NH_3_). These successive events occur at the ignition moment that takes about a fraction of second. These gasses are generated in the heart of the material particles and outwardly escape in an explosive manner at high temperature of the combustion moment. Such an extremely strong, outward stream of gasses can cause the produced particulate BCN, which has a layered structure with weak bonding, to be opened and unfolded. In fact, opening of the layered material by means of the outward gas rush causes the “gaseous unfolding” phenomenon. As evidence of this mechanism, there are a few closed, hollow and semi-hollow shells together with semi-unfolded shells (Fig. 6) remaining among the unfolded sheets. Indeed, particles convert sequentially into semi-hollow shells, hollow shells, semi-unfolded shells and fully unfolded nanosheets. Figure 7 shows a scheme exhibiting the procedure steps and summarizes the mechanism of the BCN nanosheet formation during the gaseous unfolding process. A small amount of non-fully opened sheets remaining among unfolded ones after combustion may be due to the very short duration of the ignition moment. Figure 8(a) shows one of these closed, hollow shells in the HRTEM image that remained un-opened after combustion. The hexagonal crystal habit of the boron carbonitiride can be clearly observed in Fig. 8(a), confirming the synthesis of h-BCN. The BCN nanosheets are well crystallized, as illustrated by highly ordered lattice fringes (Fig. 8(b–d)). The layered feature of the nanosheets is observed in these typical edges. The spacing between adjacent fringes is about 0.34 nm, which is very close to the calculated *d* by XRD measurements for (002) crystal plane. Alongside the (002) planes, (101) planes with a *d* spacing of ~0.21 nm can be detected in a bent edge in Fig. 8(d) due to the slight folding of the layers in the edge.

**Figure 9:**
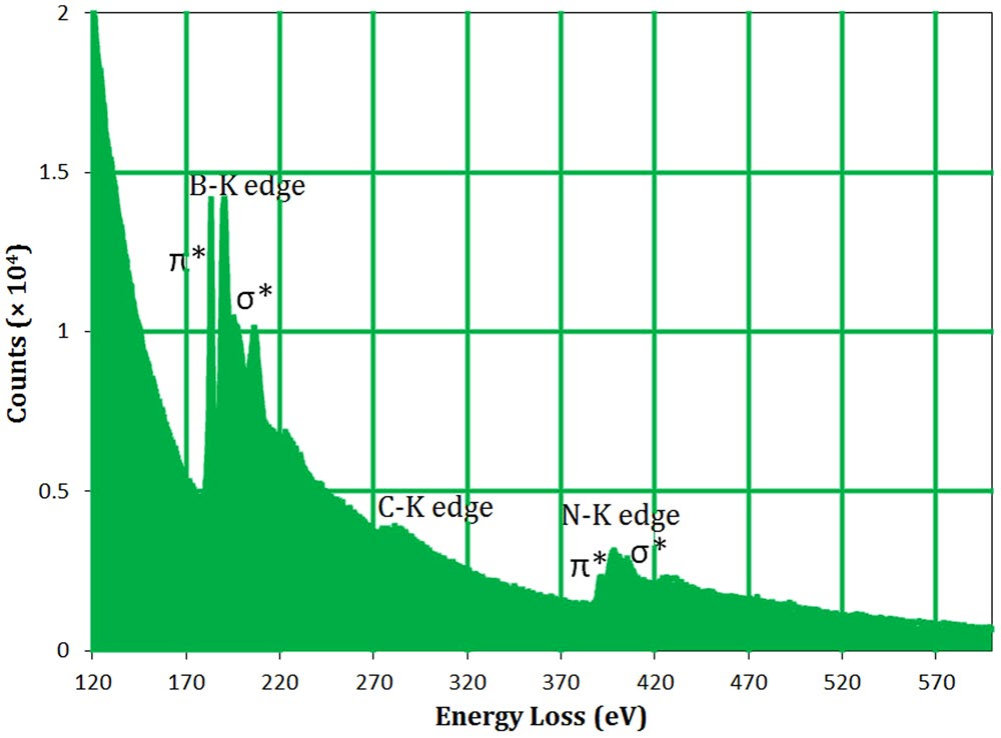
EELS spectrum of the BCN nanosheets.

EELS measurements were carried out in the high resolution electron microscope to determine the chemical stoichiometry, purity and structure of BCN nanosheets. The representative EEL spectrum depicted in Fig. 9 clearly exhibits the distinct absorption peaks located at ~190, 283 and 400 eV, which correspond to the K-shell ionization edges of B, C and N, respectively^2, 10, 19^. This result confirms the incorporation of carbon in the BN lattice, forming a B_*x*_C_*y*_N_*z*_ material. It has been reported that the B-K and N-K edges will be similar when the structure of BN is amorphous. On the other hand, these edges differ in shape considerably for h-BN^63^, which was also observed in the present work. Two sharp peaks can be detected on the left and right sides of all three edges (particularly for the B-K and N-K edges), which corresponds to 1 s → π* and 1 s → σ* antibonding orbital transition, respectively. The presence of these sharp peaks at each core-edge fine structure is typical of the sp^2^-hybridized BCN with a layered hexagonal structure^10^. It can be seen that the B-K excitation edge shows a stronger π* peak than σ*, indicating the highly crystalline nature of the material, which is consistent with the high crystallinity observed in the XRD analysis. The absence of O-K edge near 540 eV implies the purity of BCN nanosheets, although XPS analysis showed a peak related to the oxygen. This might be because XPS is a highly oxygen-sensitive technique and can easily detect oxygen from the atmosphere or adsorbed moisture^19^. By quantifying the EELS measurements, the average chemical composition of the boron carbonitride was obtained to be BC_0.2_N. This atomic ratio illustrates the nature of carbon-lean BCN, which is in a reasonable agreement with the XPS result (BC_0.3_N_1.08_). The slightly higher amount of carbon measured by XPS may be from carbonaceous contaminations that are easily detected by the XPS. Almost similar results were obtained for EELS measurements performed on several sheets, suggesting a uniform and pure hexagonal BCN structure.

### Thermal stability and optical properties

**Figure 10:**
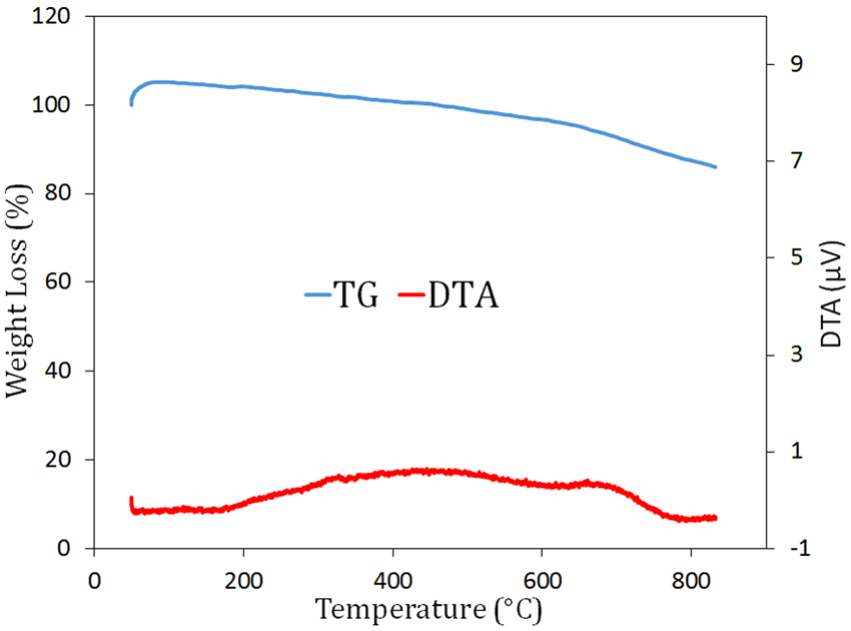
TG and DTA diagrams of the prepared sample M23 after combustion and the leaching treatment.

The oxidation resistance of sample M23 was investigated through thermal analysis up to 850 °C under air atmosphere. The thermogravimetric analysis (TGA) and differential thermal analysis (DTA) plots are shown in Fig. 10. As seen, a total weight loss of ca. 15% occurred while heating the sample from room temperature up to 850 °C. The reported weight loss may have two causes: (1) the elimination of water and carbonaceous contaminations that are physically or chemically adsorbed to the powder, and (2) the removal of a fraction of carbon atoms from the structure due to the slight and gradual oxidation of carbon into CO/CO_2_ during the heating. This low weight loss indicates the potential of the produced boron carbonitride to be considered a thermally stable nanomaterial at relatively high temperatures. The DTA diagram shows no detectable exotherm or endotherm, confirming that no major chemical reaction takes place at a single given temperature.

**Figure 11:**
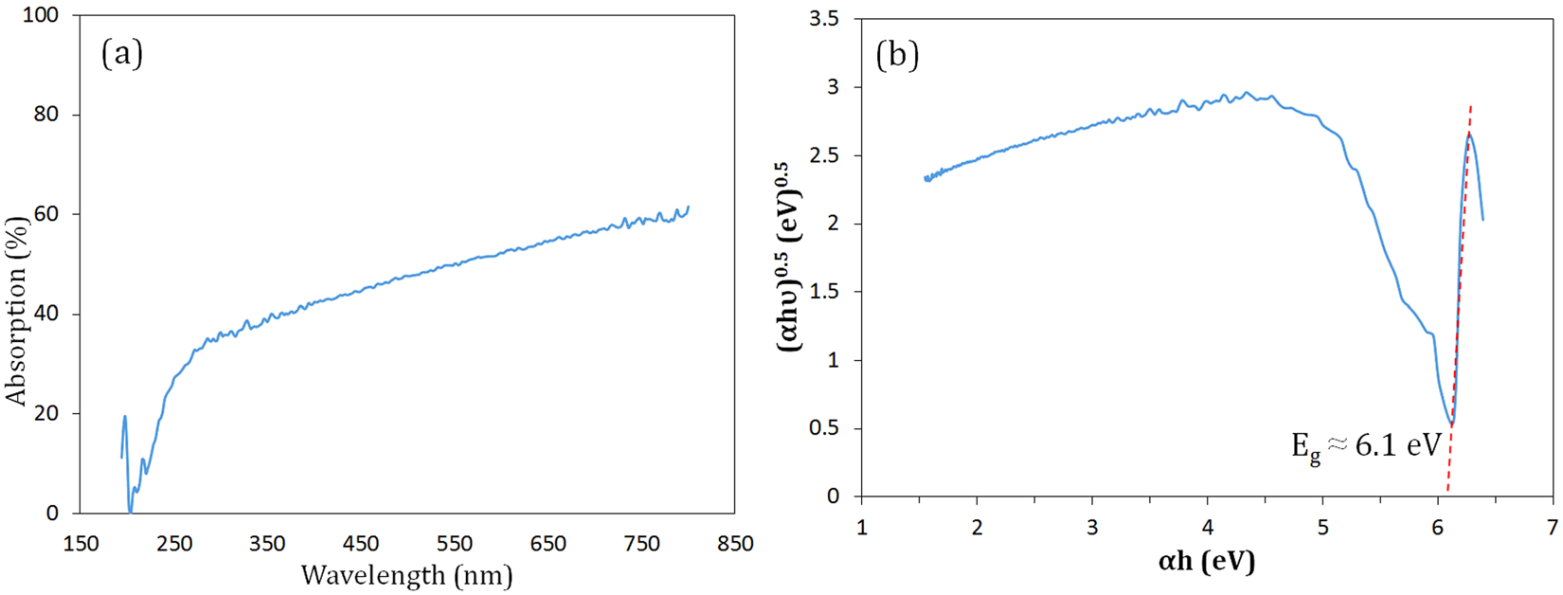
(**a**) UV-vis absorption spectrum of sample M23 after combustion and the leaching treatment. (**b**) Plot of (*α*hυ)^0.5^ versus *hυ* to yield the band gap energy.

The optical properties of sample M23 were characterized by UV-vis in diffuse reflectance spectroscopy (DRS) mode, and the results are shown in Fig. 11. Using optical absorption, the band gap of the prepared BCN nanosheets can be calculated using Tauc method^64^. A sharp absorption peak is observed at approximately 200 nm in Fig. 11(a). The absorption coefficient (*α*) for interband transition is given by the Tauc equation as follows:

11 $$\alpha h\upsilon =A{(h\upsilon -{E}_{g})}^{n}$$

where *hυ* is the photon energy and *A* is a constant. The exponent ‘*n*’ depends on the type of transition, and it may have values of 0.5, 1.5, 2, and 3, with the value of 2 exhibiting the best result in our case. The optical band gap of BCN nanosheets can be obtained by plotting (*αhυ*)^0.5^ versus *hυ*, where the intersection point of linear portion of the curve with *x*-axis yields the band gap energy. According to Fig. 11(b), the band gap energy was shown to be ~6.1 eV. This high value is an indicator of the low amount of carbon in the structure because increasing carbon content, which makes the material more similar to graphene, gives rise to the decreased band gap energy. There are several reports regarding optical properties of BN, indicating that its band gap energy (E_g_) ranges between 3.6 to 7.1 eV depending on various structures of the synthesized materials^65^. However, there are insufficient experimental studies determining E_g_ for boron carbonitride powder. In a few studies^8, 66, 67^, the authors demonstrated that their nominal BCN materials had two band gaps: the first at low value (1.2–2.8 eV) is assigned to the graphene, and the second at higher value (4.9–5.8 eV) is related to the boron nitride. Indeed, this double band gap was due to the nature of their BCN samples, which were composed of separated BN and graphene domains (i.e. BN–C composites), each having its specific band gap. In contrast, BCN thin films prepared by Tay *et al*.^68^ and Sulyaeva *et al*.^69^ showed single E_g_ values of 5.9 and a maximum of 4.5, respectively. They indicated that their products were created due to the substitutional doping, and there were no separated domains of C and BN. Therefore, our single band gap obtained from Tauc curve (Fig. 11(b)) is further evidence for the intermixing of boron, carbon, and nitrogen to form a real BCN nanostructure.

## Conclusion

The synthesis of boron carbonitride (BCN) by a magnesiothermic reduction performed in a mechanically induced self-sustaining reaction (MSR) was successfully reported. Magnesium reduced B_2_O_3_ in the presence of C_3_N_6_H_6_ after a short duration (40 min) of mechanical milling to induce a combustion reaction. Various characterizations, including XRD, FTIR, Raman, XPS, HRTEM, EELS, thermal analysis and UV-vis spectrometry, revealed various aspects of the nanomaterial. It was shown that BCN possessed a nanosheet morphology with a crystallite size of less than 7 nm and a *d*-spacing of ~0.34 nm. The removal of a large volume of gaseous species from interior particles in an explosive manner gave rise to the material unfolding into a layered nanosheet morphology. The BCN was identified to be an intermixed compound of boron, carbon and nitrogen rather than separated graphene and BN domains. The BCN nanomaterial was demonstrated to be thermally stable and had a band gap energy of ~6.1 eV.
